# Tolerance and Oncological Outcomes of In-Field Reirradiation for Locally Recurrent Breast Cancer: A Long-Term Single-Center Experience

**DOI:** 10.3390/cancers15184515

**Published:** 2023-09-12

**Authors:** Jérémy Baude, Rémi Dendale, Kim Cao, Alain Fourquet, Youlia Kirova

**Affiliations:** 1 Department of Radiation Oncology, Institut Curie, 75005 Paris, France; jbaude@cgfl.fr (J.B.); remi.dendale@curie.fr (R.D.); kim.cao@curie.fr (K.C.); alain.fourquet@curie.fr (A.F.); 2 UFR Santé, Versailles Saint-Quentin-en-Yvelines University, 78180 Saint Quentin-en-Yvelines, France

**Keywords:** breast cancer, radiation therapy, recurrence, reirradiation, toxicity, efficacy

## Abstract

**Simple Summary:**

Breast reirradiation could represent a valuable option for local recurrence of breast cancer in previously irradiated sites. We aimed to report on the efficacy and tolerability of this treatment in our institution. The results of this series combined with those available in the literature indicate that breast/chest wall reirradiation is feasible with good oncological results and low toxicity rates.

**Abstract:**

Background: The management of cancer relapse in previously irradiated tissues is a challenging therapeutic issue. The aim of this work was to report our experience with breast reirradiation for locoregionally recurrent breast cancer. Methods: All patients who underwent breast or chest wall in-field reirradiation at the Institut Curie, Paris, France, between 2003 and 2019, were identified. Efficacy outcomes and physician-reported toxicities were retrospectively assessed. Results: A total of 21,372 patients underwent breast irradiation in our institution. Of these, 28 received a second course of radiotherapy to the homolateral breast/chest wall. A total of 18 (64%) patients were treated with a curative intent, and 10 (36%) were treated for palliative purposes. Only one acute and one late grade 3 adverse events were reported. One patient with major cardiovascular risk factors died of myocardial infarction 13 months after left breast reirradiation. The 2-year LRFS, OS, DSS, PFS and MFS were 59%, 79%, 82%, 46% and 75%, respectively, in the whole cohort. The 2-year LRFS (72% vs. 31%, *p* = 0.02), OS (94% vs. 50%, *p* < 0.01), DSS (94% vs. 56%, *p* < 0.01) and PFS (61% vs. 20%, *p* = 0.02) differed significantly between patients treated with curative or palliative intent but not the MFS (78% vs. 69%, *p* = 0.77). Among the patients, eight (29%) remained relapse-free 5 years after reirradiation. Conclusion: Breast/chest wall reirradiation appears to be feasible with good disease control, especially in patients treated with a curative intent, and presents acceptable toxicity rates.

## 1. Introduction

In recent years, improved management of localized and locally advanced breast cancer has led to a decreased rate of locoregional cancer recurrence after breast-conserving surgery followed by adjuvant radiation therapy (RT). However, approximately 8% of patients still develop an ipsilateral breast relapse [1]. Treatment of this recurrence in previously irradiated sites is intricate and often relies on total mastectomy and systemic treatments [2].

Reirradiation could represent a valuable therapeutic option in this setting, especially in patients who seek second partial breast surgery but also in patients who require palliative local treatment. In both cases, reirradiation raises a number of issues concerning its feasibility, efficacy and toxicity [3]. Therefore, the selection of eligible patients remains a challenge and often relies on a case-by-case medical collegial decision. Different doses, RT techniques, and radiation fields have been previously reported. Since the first series published in the literature showed positive signals for local reirradiation [4,5], systemic treatments as well as RT techniques have evolved greatly. More recently, some have studied the role of reirradiation with concurrent hyperthermia [6,7,8], while others have found favourable results with partial external beam reirradiation [9,10]. Proton therapy is another investigated therapeutic weapon [11,12]. Although efficacy outcomes are excellent, with acceptable toxicity, only short-term results are currently available. On the other hand, Hannoun-Levi et al. recently reported long-term outcomes of a second conservative treatment consisting of a lumpectomy and breast brachytherapy: toxicity was low and 10-year DFS was 78% [13].

However, most of these techniques evaluated (hyperthermia, proton therapy, brachytherapy) are only available in a limited number of tertiary facilities, and data on “regular” radiation therapy, defined as feasible in most radiotherapy centres, are still scarce.

Therefore, the purpose of this study was not only to evaluate the toxicity and the feasibility of in-field reirradiation in real-life patients, but also to report the long-term results of this treatment option, in the modern era of systemic and targeted therapies. We also provide additional data about locoregional recurrences and their management in a French single centre.

## 2. Materials and Methods

### 2.1. Patients

This retrospective study was conducted at the Institut Curie, Paris. It included adult patients who underwent two courses of RT delivered to the breast/chest wall (in-field reirradiation) ± lymph nodes with curative or palliative intent between 2003 and 2019. Those who underwent reirradiation only in volumes other than the ipsilateral breast/chest wall were excluded for the purpose of relevant toxicity evaluation.

### 2.2. Data Extraction and Collection

A complete medical search for all patients who fit the inclusion criteria was performed within our institution. They were further classified according to whether the purpose of reirradiation was curative or palliative.

Physician-reported acute (during radiotherapy and the first 6 months following the end of RT) and late (later than 6 months after RT) toxicities were assessed using the common terminology criteria for adverse events (CTCAE) 5.0.

Efficacy outcomes were evaluated through local recurrence-free survival (LRFS), progression-free survival (PFS), overall survival (OS), disease-specific survival (DSS), and metastasis-free survival (MFS). Progression-free survival was defined as the period of time from reirradiation to disease recurrence, progression and death. Overall survival was the time from reirradiation to death of any cause. Disease-specific survival (DSS) accounted for the time from the second course of RT to death from breast cancer. Local recurrence-free survival (LRFS) and metastasis-free survival (MFS) were defined as the time from treatment to local recurrence and metastatic relapse, respectively. Progression was defined as the earliest evidence of radiological progression or clinical progression according to the occurrence or reappearance of disease-related symptoms.

When RT was delivered in a hypofractionated regimen, the equivalent dose in 2 Gy (EQD2, in Gy_2_) was calculated using an α/β = 3 Gy [14]. The cumulative dose was defined as the sum of the highest dose prescribed in each RT course, including the boost.

### 2.3. Statistical Analysis

Descriptive analyses were performed using medians with interquartile ranges (IQR) for quantitative variables and percentages for qualitative variables. Survival rates and medians were determined using the Kaplan–Meier method. Survival curves were compared with the Grehan–Breslow–Wilcoxon test. Median follow-up was determined using the reverse Kaplan–Meier method. Tests were two-sided. *p* values less than 0.05 were considered significant. Analyses were performed using GraphPad Prism 9.4 software.

## 3. Results

### 3.1. Description of the Cohort

**Figure 1:**
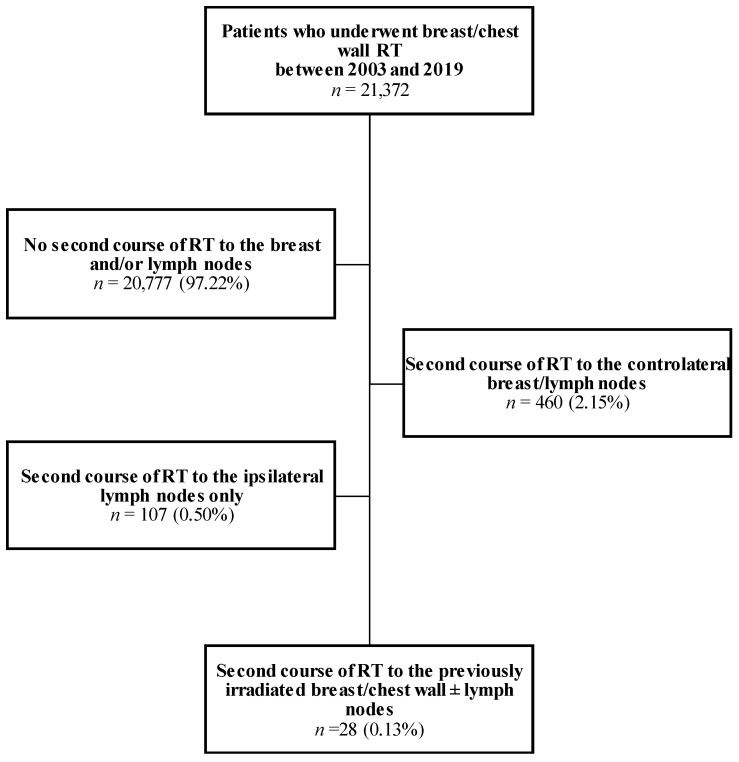
Flow chart of the studied population. RT: radiotherapy.

Between 2003 and 2019, we identified 21,372 patients who underwent breast or chest wall irradiation with or without lymph nodes in our institution. Of these, 460 (2.15%) received a second irradiation to the contralateral breast and/or lymph nodes, 107 (0.5%) to the homolateral lymph nodes without breast/chest wall reirradiation because only regional recurrence occurred in patients already treated locally, and only 28 (0.13%) received a second course of in-field reirradiation to the homolateral breast or chest wall (Figure 1). These 28 patients were included in this study. All of them were female.

#### 3.1.1. Patient Characteristics at First Irradiation

Patient characteristics at first irradiation are given in Table 1. The median age was 57 (IQR, 44–70.5) years. Before radiotherapy, seven (25%) women underwent total mastectomy, and 18 (61%) underwent lumpectomy. Four (14%) patients had only a biopsy, as one had her first course of RT given for postmastectomy recurrence at 19 years, one refused surgery, one was rejected for surgery because of age (80 years old) and received hormone therapy and RT only, and one underwent palliative RT. Radiotherapy was normofractionated in all patients. The standard protocol was 50 Gy/25 fractions to the chest wall +/− lymph nodes or 50 Gy/25 fractions to the breast, followed by a sequential normofractionated boost to the tumor bed. The median dose was 56 (range, 50–66) Gy_2_, including the boost. A total of 14 patients received a sequential boost of 10 Gy in 5 fractions (*n* = 2), 16 Gy in 8 fractions (*n* = 10) and up to 20 Gy/10 fractions in two patients with advanced disease.

#### 3.1.2. Patient Characteristics at Second Irradiation

Patient characteristics at the second irradiation are given in Table 1. The median age was 63 (IQR 49–78) years. Overall, three (11%) women presented with multiple recurrences before the second course of in-field radiation therapy. A total of 20 (71%) patients underwent salvage surgery before radiation therapy: of these, 14 (50%) had a total mastectomy, 6 (21%) of them underwent a partial breast resection and 3 (11%) had a biopsy only. Of those who underwent biopsy only, 2 had a unique gross nodule biopsy-resection and were subsequently treated for curative purposes. Five (18%) women had a clinical diagnosis of recurrence and were treated with curative intent. The median time between RT courses was 47 (22.75–109.5) months. RT was given for curative and palliative purposes to 18 (64%) and 10 (36%) patients, respectively. A total of 10 patients (36%) presented with a large residual disease before radiation therapy, 9 of whom were treated with palliative intent. The clinical target volume (CTV) included chest wall ± lymph nodes in 20 (71%) patients, whole breast ± lymph nodes in 7 (25%) and partial breast in 1 (4%). Normofractionated RT was given to 26 (93%) patients with a median dose of 48 (30–50) Gy, including boost. 

Two patients received hypofractionated radiation therapy (2 × 6.5 Gy and 5 × 4 Gy). One patient received a sequential boost of 16 Gy (8 × 2 Gy). The median dose was 47 (30–50) Gy_2_, including the boost. The median cumulative dose was 99 (90.6–114.3) Gy_2_.

### 3.2. Reirradiation Toxicity Outcomes

**Figure 2:**
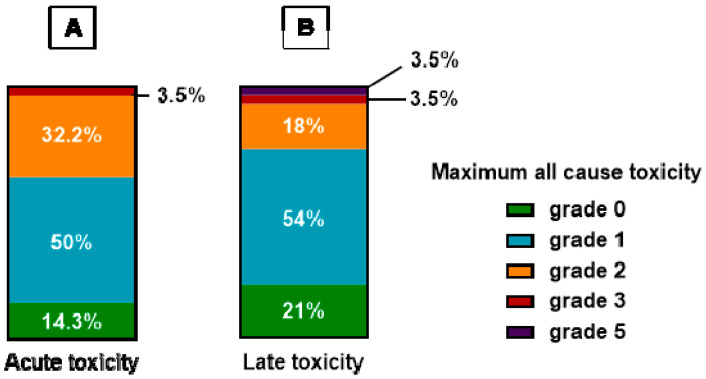
Maximum all-cause acute (**A**) and late (**B**) physician-reported toxicity after breast/chest wall reirradiation. Toxicity was evaluated according to the common terminology criteria for adverse events (CTCAE) 5.0.

The maximum grade of observed acute toxicity described was grade 1 in 14 (50%) patients, grade 2 in 9 (32.2%) and grade 3 in only one (3.5%) patient, as shown in Figure 2A. No grade 4 acute toxicity was found in our series. The most common adverse events (AEs) were radiodermatitis and pain, which were observed in 24 (85.7%) and 11 (39%) patients, respectively. The maximum grade late toxicity was grade 1 in 15 (54%) patients, grade 2 in five (18%), grade 3 in one patient (3.5%) and grade 5 in one (3.5%). The results are given in Figure 2B. The side effects were mostly skin fibrosis and skin telangiectasia. There was no brachial plexopathy, lung fibrosis or lymphedema in these series.

The patient reported with grade 5 toxicity was a patient with multiple cardiovascular risk factors (unbalanced type 2 diabetes, severe obesity (BMI = 33.7 kg/m^−2^), treated hypertension and treated dyslipidemia) who died of myocardial infarction 13 months after left breast reirradiation for palliative purposes. The first RT course dose to the breast was 50 Gy with an additional sequential boost of 16 Gy, but this 3D conformal irradiation was realized in the lateral position, and the dose to the heart was close to 0 Gy. The patient also received a 3D conformal regional lymph node irradiation. Reirradiation to the chest wall and axilla was realized using the IMRT technique by tomotherapy and delivered 30 Gy/15 fractions, bringing the cumulative dose to 96 Gy_2_. (Mean heart dose = 6.86 Gy) The period between the first and second irradiations was 111 months. Systemic treatments consisted of adjuvant FEC75 (fluorouracil, epirubicin, cyclophosphamide) at initial management followed by 5 years of tamoxifen and post-RT docetaxel and fulvestrant at relapse.

### 3.3. Reirradiation Efficacy Outcomes

**Figure 3:**
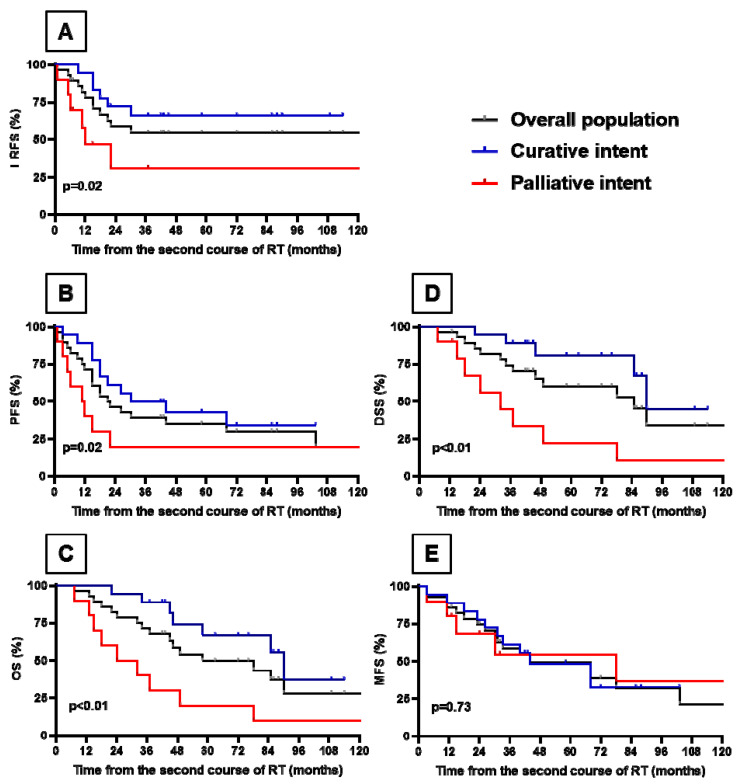
Kaplan–Meier estimates of LRFS (**A**), PFS (**B**), OS (**C**), DSS (**D**) and MFS (**E**) in the whole cohort and in patients treated with curative and palliative intent. Survival rates were determined using the Kaplan–Meier method. Survival curves of patients treated with palliative (*n* = 10) and curative (*n* = 18) intent were compared using the Grehan–Breslow-Wilcoxon test. RT: radiotherapy, LRFS: local relapse-free survival, PFS: progression-free survival, OS: overall survival, DSS: disease-specific survival, MFS: metastasis-free survival.

The median follow-up from completion of the in-field reirradiation was 45.5 (range, 33.5–79.8) months. In the whole cohort, the 2-year LRFS (A), PFS (B), OS (C), DSS (D) and MFS (E) were 59%, 46%, 79%, 82% and 75%, respectively (Figure 3). Two-year LRFS was significantly better in patients treated with curative intent than in those treated with palliative intent (72% vs. 31%, *p* = 0.02), as was the 2-year PFS (61% vs. 20%, *p* = 0.02), as well as the 2-year OS (94% vs. 50%, *p* < 0.01) and the 2-year DSS (94% vs. 56%, *p* < 0.01). However, the 2-year MFS was not significantly different between the groups (78% vs. 69%, *p* = 0.77). Furthermore, we did not find any significant difference in LRFS depending on the efficacy of the first course of RT or the time between the first RT course and reirradiation (Supplementary Figures S1 and S2). Among patients treated in a palliative setting (*n* = 10), 7 (70%) had a clinical improvement after the completion of reirradiation. 

Among all women, 8 (29%) remained relapse-free 5 years after the second course of in-field radiation therapy. During the follow-up period, we observed 11 local and 11 metastatic recurrences. Individual data about treatments and outcomes are available in Table 2.

## 4. Discussion

This single-centre series has shown that with decreasing recurrence, reirradiation of the treated volumes is a rare condition, but it represents a feasible treatment option with an acceptable rate of side effects and could improve patient outcomes. 

Even though approximately 3–8% of patients with breast cancer eventually develop an ipsilateral breast recurrence [1], we only identified 28 patients (0.13%) among the 21,372 who received a first breast irradiation in our institution and who underwent in-field reirradiation for their breast or chest wall recurrence. Reirradiation is a heterogeneous and centre-dependent practice [3], and the management of local recurrence relies mainly on surgery in our institution. Recently, the Italian Association of Radiotherapy and Clinical Oncology (AIRO) found that only a third of radiation therapy centres in Italy [15] propose breast/chest wall reirradiation, highlighting concerns about its feasibility, efficacy and toxicity.

In our series, we report favourable outcomes for reirradiation, similar to other retrospective studies obtained with various radiation therapy regimens, doses and techniques, with 58.9% LRFS and 81.6% OS at 2 years. As expected, the results were particularly satisfactory in women treated for curative purposes, with a 2-year LRFS of 72% and a 2-year OS of 94%. These results were comparable to those found by Fattahi et al. in 72 patients [6]. The 2-year LRFS was 93.1%, and the 2-year OS was 76.8% for those treated with curative intent without gross disease at reirradiation and 57.1% and 71.4% in those treated with curative intent with gross disease [6]. 

A further noteworthy result was the identification of 8 (29%) long-term responders, with no recurrence at 5 years after the second radiation therapy. This suggests that some patients may particularly benefit from reirradiation, raising the issue of patient selection. Identifying predictive factors of response to reirradiation seems important, but data available in the literature on reirradiation are mainly based on modest, heterogeneous retrospective series, making subgroup analyses complex. There is mostly reported experience of partial breast irradiation in cases of recurrence. One of the largest reported series is the GEC ESTRO experience, reported by Hannoun-Levi et al., who retrospectively gathered data across seven European centres from patients who underwent a second conservative treatment combining lumpectomy and accelerated partial breast reirradiation (APBrI) using brachytherapy [16]. In the 508 included patients, APBrI was effective and well tolerated, and GEC-ESTRO APBI classification was recorded as an independent prognostic factor. Tumor size (>20 mm) may also represent another valuable prognostic factor [7,17]. These findings may be useful to patients and physicians for choosing between conservative and radical treatments in the decision-making process.

Different approaches are currently proposed to improve the outcomes and toxicity of patients who require reirradiation. The NRG Oncology/RTOG 1014 phase 2 trial demonstrated that partial breast reirradiation delivering 1.5 Gy twice daily for 15 days (45 Gy) was associated with low rates of locoregional recurrence and toxicity in 58 patients [2]. Hyperthermia combined with RT may also be an interesting treatment for locally recurrent breast cancer [18]. A meta-analysis from Datta et al. showed that the combination improved the complete response compared to RT alone [19]. Proton reirradiation could also represent a valuable option, with good outcomes and acceptable toxicity [12,20]. Despite the accumulation of data, the best RT regimen and technique to propose for breast/chest wall reirradiation remains unknown. Further prospective data are expected from various studies currently underway to clarify the grey areas [21,22,23].

In the presented series, one patient died from myocardial infarction during the follow-up period. This patient was heavily pretreated for bilateral recurrent breast cancer and presented with multiple comorbidities and factors at risk. As this death occurred after left breast reirradiation (mean heart dose = 6.86 Gy), we reported her case as grade 5 treatment-related toxicity, even though it may be at least in part explained by other confounding factors, as the patient had multiple cardiovascular risk factors and had received antracycline-based chemotherapy. It is well known that cardiac toxicity is very complex and multifactorial [24]. In the case of this patient, the first breast irradiation was realized in the lateral position using a specific technique, as previously described, and the heart dose was close to 0 Gy [25]. This technique of whole breast radiotherapy in the lateral decubitus position for patients with large breasts and early-stage breast cancer provides an excellent dosimetric profile, with low doses to the heart and ipsilateral lung. It is also very well tolerated, with a good acute toxicity profile, and was chosen in the case of this patient because of her comorbidities.

In the reported series, the other patients experienced a low rate of acute and late AEs, with 3.5% of acute and late grade 3 AEs and mainly skin toxicity. Given the small number of events, a search for toxicity-associated factors was not possible. Toxicity rates vary greatly in the literature [3], depending on the characteristics of reirradiation (particle used, irradiated sites, dose, etc.), with grade 3 AEs ranging between 0% [9] and 30% [18] between series. Comparisons between cohorts are difficult, but altogether, these data suggest that reirradiation is feasible.

This work has several limitations, mainly due to the small size and heterogeneity of the population. Some patients were treated with breast-conserving surgery, and others were treated with mastectomy. Another limitation is the retrospective nature of our study. At the same time, these series with long-term results show the feasibility of in-field reirradiation, even when patients were treated with large target volumes. With the modern techniques of radiation therapy, better results can be expected. 

These real-life data add to the growing body of evidence supporting that reirradiation may be an appropriate treatment for patients requiring locoregional treatment in previously irradiated sites and could be a new chance to cure these patients. This treatment option could be considered an individualized treatment for many patients with locally recurrent breast cancer.

## 5. Conclusions

Breast or chest wall reirradiation appears to be feasible with good disease control, especially in patients treated with a curative intent, and acceptable toxicity rates. In addition, a significant number of patients were disease-free 5 years after the second course of RT. Prospective larger data with longer follow-up are needed to confirm these findings.

## Figures and Tables

**Table 1:** Patients and treatment characteristics for the first and second RT courses.

*n* = 28	First RT Course	Second RT Course
Age (years), median (IQR)	57 (44–70.5)	63 (49–78)
Primary surgery (%)		
Mastectomy	6 (22%)	14 (50%)
Lumpectomy	18 (64%)	6 (21%)
Biopsy only	4 (14%)	3 (11%)
No surgery	0	5 (18%)
Resection status (%)		
R0	21 (75%)	14 (70%)
R1	2 (7%)	3 (15%)
NR	5 (18%)	3 (15%)
Axillary surgery (%)		
Axillary lymph node dissection	18 (64%)	8 (29%)
Sentinel lymph node biopsy	4 (14%)	3 (11%)
No axillary surgery	6 (22%)	17 (61%)
Histology (%)		
DCIS	0	0
Invasive ductal carcinoma	22 (79%)	15 (54%)
Invasive lobular carcinoma	5 (18%)	2 (7%)
Invasive micropapillary carcinoma	1 (4%)	5 (18%)
Clinical TNM classification (%)		N/A
cTx	1 (4%)	
cT1	3 (11%)	
cT2	15 (54%)	
cT3	2 (7%)	
cT4	7 (25%)	
cN0	17 (61%)	
cN1	10 (36%)	
cN2	1 (4%)	
cN3	0	
Pathological TNM classification (%)		N/A
pT0	2 (7%)	
pT1	12 (43%)	
pT2	7 (25%)	
pT3	0	
pT4	2 (7%)	
N/A	5 (18%)	
pN0	12 (43%)	
pN1	8 (29%)	
pN2	3 (11%)	
pN3	0	
No axillary surgery	5 (18%)	
AJCC 8th edition stage (cTNM) (%)		N/A
I	4 (14%)	
II	16 (57%)	
III	8 (29%)	
IV	0	
Hormone receptor and HER2 status (%)		
HR+ HER2- (%)	16 (57%)	14 (50%)
HR+ HER2+ (%)	1 (4%)	2 (7%)
HR- HER2+ (%)	3 (11%)	2 (7%)
TN (%)	3 (11%)	2 (7%)
NR (%)	5 (18%)	8 (29%)
Chemotherapy (%)		
Neoadjuvant	8 (29%)	2 (7%)
Adjuvant	6 (21%)	14 (50%)
Both	4 (14%)	0
Without surgery	0	1 (4%)
None	10 (36%)	11 (39%)
Hormone therapy (%)		
Yes	19 (68%)	18 (64%)
None	9 (32%)	10 (36%)
Radiotherapy modality (%)		
Photons	4 (14%)	8 (29%)
Electrons	1 (4%)	13 (46%)
Photons + electrons	12 (43%)	6 (21%)
60Cobalt	3 (11%)	1 (4%)
60Cobalt + electrons	5 (18%)	0
NR	3 (11%)	0
Radiotherapy fields (%)		
Chest wall	6 (21%)	20 (71%)
Whole/partial breast	22 (79%)/0	7 (25%)/1 (4%)
Berg II-IV	22 (79%)	6 (21%)
Internal mammary chain	17 (61%)	5 (18%)
Axillary region (Berg I)	7 (25%)	2 (7%)
No lymph node irradiation	6 (21%)	21 (75%)
Radiotherapy positioning (%)		
Dorsal decubitus	16 (57%)	24 (86%)
Lateral decubitus	3 (11%)	2 (7%)
NR	9 (32%)	2 (7%)
Use of boost (%)	14 (50%)	1 (3.5%)
RT dose with boost (Gy_2_), median (IQR)	60 (50–66)	48 (30–50)
Time from first RT course (months), median (IQR)	47 (22.75–109.5)	
Cumulative RT dose (Gy_2_), median (IQR)	99 (90.6–114.3)	

NR: not reported, N/A: not applicable, DCIS: ductal carcinoma in situ, HR: hormone receptors, TN: triple negative.

**Table 2:** Individual characteristics of patients: treatments and outcomes.

Patient No.	Age at 2nd RT Course	Year of 2nd RT Course	Time from 1st RT Course (Months)	Site and Dose of Reirradiation (Gy)	Cumulative Dose to the Breast/Chest Wall (Gy_2_)	Treatments Besides RT	Intent	PFS (Months)	Progression after 2nd RT Course	OS (Months)
1	38	2003	31	Chest wall 38	104	Mastectomy + adjuvant Capecitabine + Triptorelin + Letrozole	Palliative	3	Unique choroid metastasis	15
2	80	2003	24	Whole Breast 39.6	115.1	Adjuvant Vinorelbin + Methotrexate	Palliative	5	Extensive cutaneous lymphangitis	78
3	37	2003	18	Chest wall 50	110	Mastectomy + adjuvant Vinorelbine + Methotrexate + Enantone + Anastrozole	Curative	15	Breast cutaneous nodes	46
4	78	2004	4	Chest wall 30	80	Continuing Exemestane	Curative	60+ (DF)		58
5	61	2004	35	Chest wall 52, Berg I 46	118	Mastectomy + adjuvant Docetaxel + Fulvestrant	Curative	68	Multiple bone metastases	114+ (A)
6	77	2004	124	Chest wall 50, Berg II- IV 48, CMI 48, Boost to the tumor bed 16	116	Mastectomy	Curative	9	Chest wall recurrence	34
7	79	2005	11	Whole breast 20	70	Adjuvant Capecitabine + Tamoxifen	Palliative	1,0	Out-of-field breast cutaneous nodes	32
8	54	2005	98	Chest wall 48	98	Partial mastectomy	Palliative	203+ (DF)		203+ (A)
9	83	2005	10	Whole breast 13 (2 × 6.5)	86.5	Concurrent and adjuvant Exemestane	Palliative	126	Metastatic pleurisy	7
10	60	2005	45	Chest wall 46	110	Partial mastectomy + adjuvant Docetaxel + 5FU + Letrozole	Curative	26	Controlateral axillary adenopathies	90
11	45	2008	130	Chest wall 20	70	Mastectomy + axillary lymph node resection + adjuvant Docetaxel + Bevacizumab	Curative	3	Inguinal adenopathy	22
12	78	2009	14	Whole breast 10 (2 × 5)	49.6	Adjuvant Navelbine + Trastuzumab	Palliative	12	Breast cutaneous nodes	24
13	73	2009	19	Chest wall 48	114	Neoadjuvant Paclitaxel + Bevacizumab + mastectomy + axillary lymph node resection	Palliative	11	Lung metastases and multiple mediastinal adenopathies	18
14	64	2010	94	Chest wall 48	114	Mastectomy + adjuvant Docetaxel + Cyclophosphamide	Palliative	15	Lung metastatis	37
15	89	2010	67	Chest wall 20	77.6	Partial mastectomy + adjuvant Exemestane	Curative	44	Unique intramuscular metastasis of the trapezius	45
16	78	2010	91	Chest wall 50	115	Partial mastectomy + adjuvant Anastrozole	Curative	15	Unique infield presternal cutaneous node	42+ (A)
17	75	2011	109	Chest wall 50, Berg II- IV 46, CMI 48	101	Mastectomy + adjuvant FEC100 then Docetaxel + Letrozole	Curative	72+ (DF)		72 (A)
18	61	2012	111	Whole breast 30	96	Axillary lymph node resection + adjuvant Docetaxel + Fulvestrant	Palliative	6	Breast recurrence	13 *
19	37	2013	34	Chest wall 46	92	Mastectomy + sentinel lymph node biopsy + adjuvant Docetaxel + Cyclophosphamide + Triptorelin	Curative	103	Multiple bone metastases	109+ (A)
20	50	2014	49	Chest wall 30, Berg II- IV 46, CMI 46	96	Mastectomy + adjuvant FEC100 then Docetaxel + Enantone + Letrozole	Curative	86+ (DF)		86+ (A)
21	51	2015	36	Chest wall 48, Berg II-IV 40, CMI 40	96	Mastectomy + axillary lymph node resection + adjuvant Trastuzumab + Pertuzumab	Curative	21	Breast recurrence and multiple mediastinal adenopathies	85
22	62	2015	92	Chest wall 45	97	Partial mastectomy + adjuvant Docetaxel + Trastuzumab + Pertuzumab	Curative	88+ (DF)		88+ (A)
23	72	2016	38	Chest wall 50	100	Mastectomy + adjuvant Letrozole	Curative	30	Chest wall recurrence	76+ (A)
24	46	2017	138	Whole breast 50	118	Adjuvant Capecitabine + Trastuzumab + Pertuzumab + Tamoxifene	Palliative	22	Breast cutaneous nodes	49
25	82	2017	149	Chest wall 48, Berg I-IV 48	98	Mastectomy + sentinel lymph node biopsy + adjuvant Exemestane	Curative	18	Chest wall recurrence	63+ (A)
26	42	2019	7	Partial breast 26	76	-	Curative	43+ (DF)		43+ (A)
27	42	2019	123	Chest wall 50	116	mastectomy + sentinel lymph node biopsy + adjuvant Tamoxifene	Curative	18	Multiple supra and infradiaphragmatic adenopathies	37+ (A)
28	71	2019	142	Whole breast 50, Berg II- IV 50, CMI 50	116	Partial mastectomy + adjuvant Letrozole	Curative	42+ (DF)		42+ (A)

* Died of myocardial infarction 13 months after RT, RT: radiotherapy, Gy: Gray, PFS: progression-free survival, OS: overall survival, DF: disease-free at last visit, A: alive.

## Data Availability

Data supporting reported results can be provided by the corresponding author upon reasonable request.

## Supplementary Materials

The following supporting information can be downloaded at https://www.mdpi.com/article/10.3390/cancers15184515/s1, Figure S1: Kaplan-Meier estimates of LRFS for reirradiation according to the efficacy of the first course of RT; Figure S2: Kaplan-Meier estimates of LRFS for reirradiation according to the time between RT courses.
