# Supplementary material for: Tolerance and Oncological Outcomes of In-Field Reirradiation for Locally Recurrent Breast Cancer: A Long-Term Single-Center Experience
Source: Cancers (Basel). 2023 Sep 12;15(18):4515. doi: 10.3390/cancers15184515 (PMC10527329; doi:10.3390/cancers15184515)
Supplement: Supplementary file 1 [file cancers-15-04515-s001.zip › cancers-2581378-supplementary.pdf]

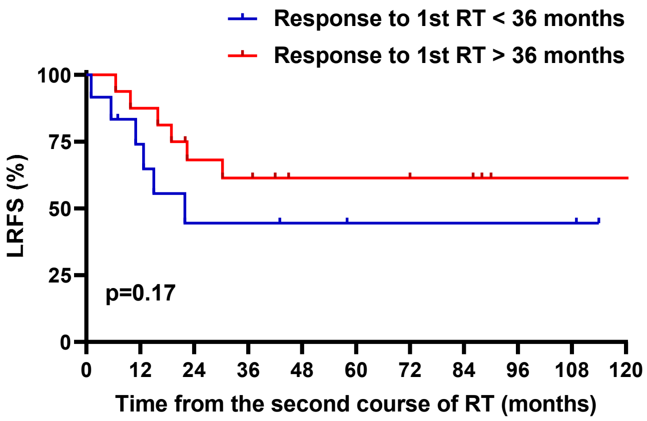

Figure S1. Kaplan-Meier estimates of LRFS for reirradiation according to the efficacy of the first course of RT
Survival rates were determined using the Kaplan-Meier method. Survival curves of patients who locally relapsed less than 36 months after their first course of RT (n=12) and those who locally relapsed beyond 36 months (n=16) were compared using the Grehan-Breslow-Wilcoxon test.
RT: radiotherapy, LRFS: local relapse-free survival.

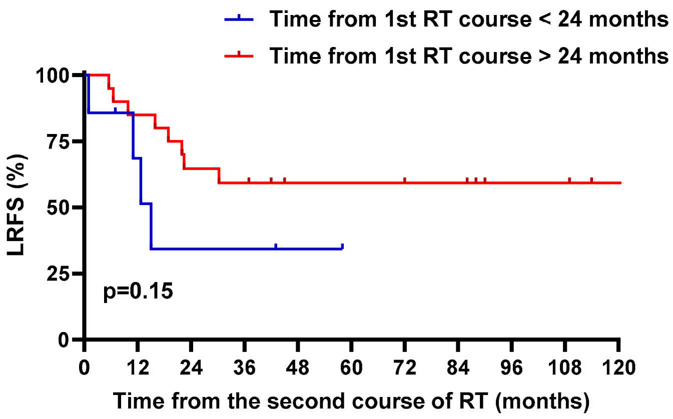

Figure S2. Kaplan-Meier estimates of LRFS for reirradiation according to the time between RT courses
Survival rates were determined using the Kaplan-Meier method. Patients were divided between those who underwent re-RT < or > 24 months after first RT course (n=7 and 21, respectively). Survival curves were compared using the Grehan-Breslow-Wilcoxon test.
RT: radiotherapy, LRFS: local relapse-free survival.
